# Immediate breast reconstruction uptake in older women with primary breast cancer: systematic review

**DOI:** 10.1093/bjs/znac251

**Published:** 2022-08-01

**Authors:** Rachel Xue Ning Lee, Maria Joao Cardoso, Kwok Leung Cheung, Ruth M Parks

**Affiliations:** Nottingham Breast Cancer Research Centre, University of Nottingham, Nottingham, UK; Queen’s Medical Centre Campus, Nottingham University Hospitals NHS Trust, Nottingham, UK; Nottingham Breast Cancer Research Centre, University of Nottingham, Nottingham, UK; Breast Unit, Champalimaud Foundation and Nova Medical School Lisbon, Lisbon, Portugal; Nottingham Breast Cancer Research Centre, University of Nottingham, Nottingham, UK; School of Medicine, University of Nottingham, Nottingham, UK; Nottingham Breast Cancer Research Centre, University of Nottingham, Nottingham, UK; School of Medicine, University of Nottingham, Nottingham, UK

## Abstract

**Background:**

Postmastectomy immediate breast reconstruction (PMIBR) may improve the quality of life of patients with breast cancer, of whom older women (aged 65 years or more) are a growing proportion. This study aimed to assess PMIBR in older women with regard to underlying impediments (if any).

**Methods:**

MEDLINE, Embase, and PubMed were searched by two independent researchers up to June 2022. Eligible studies compared PMIBR rates between younger and older women with invasive primary breast cancer.

**Results:**

A total of 10 studies (2012–2020) including 466 134 women were appraised, of whom two-thirds (313 298) were younger and one-third (152 836) older. Only 10.0 per cent of older women underwent PMIBR in contrast to 45.0 per cent of younger women. Two studies explored factors affecting uptake of PMIBR in older women; surgeon-associated (usual practice), patient-associated (socioeconomic status, ethnicity, and co-morbidities), and system-associated (insurance status and hospital location) factors were identified.

**Conclusion:**

Uptake of PMIBR in older women is low with definable (and some correctable) barriers.

## Teaser

Uptake of postmastectomy immediate breast reconstruction (PMIBR) in older women is significantly lower than in younger women and there is a paucity of data in the literature on the barriers and facilitators to PMIBR in older women. Considering the increasing proportion of older women living with breast cancer, further research is warranted.

## Introduction

Breast cancer incidence correlates with age, such that approximately half develop in older women (aged 65 years or more)^1^. Breast reconstruction may be considered less important in older than in younger women by some^2^. Indeed, the likelihood of the treating team offering breast reconstruction declines sharply for women aged 70 years or older^3^, with some centres restricting reconstructive procedures to those aged less than 65 years alone^4^. Reasons for this include concerns about complication risks or prolonged hospital stays^5–9^. Recent studies^10–13^ have suggested that older women do not have these issues. In fact, there seems to be very little difference in outcomes or complications between younger and older women for both implant^10,11^ and autologous^12,13^ reconstruction.

Postmastectomy immediate breast reconstruction (PMIBR) improves psychosocial well-being, quality of life, and body image^14–17^. Previous studies^18^ have suggested that older women are less concerned by the loss of a breast, and therefore more reluctant to undergo additional surgical procedures as well as facing demands from increased frailty^19^. Lack of awareness of options of immediate breast reconstruction (IBR) has been documented for some older patients^20–23^. Although some studies^24–26^ have suggested that there is a lower rate of PMIBR among older women, no studies have quantified the exact difference in PMIBR rates between younger and older women, or explored the underlying reasons for this. The present study aimed to address these questions.

## Methods

This systematic review was conducted in accordance with the guidelines outlined in the PRISMA statement^27^.

### Search strategy and study selection

A systematic search was carried out in MEDLINE, Embase, and PubMed databases on 5 June 2022. Reference lists of all included articles and relevant systematic reviews were also hand-searched for possible additional publications. Only full-text articles published in English and peer-reviewed journals were included.

An age cut-off of 65 years or more was applied, as this was commonly used in the medical literature to define older women^28^. The search strategy was finalized with the assistance of a clinical librarian. The following terms were used to search for titles and abstracts: ‘Breast cancer’, ‘Breast reconstruction’, and ‘Mastectomy (including terms specifying all major subgroups). Details of each search strategy for the respective databases are available in *Appendix S1*. Two independent researchers retrieved search results from the database and imported them into Mendeley reference manager. Duplicate publications were excluded from the search. Articles were screened by the same independent researchers in two stages. Titles and abstracts were screened to assess their potential relevance for full review, then relevant full texts of potentially relevant articles were retrieved and screened. Any discrepancies were resolved through discussion with a third reviewer. The reference lists of all relevant studies were also screened to ensure no study had been missed. In accordance with the PRISMA guidelines, a flow diagram was developed to report the process of study selection.

The following inclusion criteria were used: female participants; studies that compared older women (aged 65 years or more) with a cohort of younger women, or included women of multiple age groups with clear representation of those aged at least 65 years; studies with participants who had undergone mastectomy and IBR for primary breast cancer.

The following exclusion criteria were applied: studies that did not fulfil the inclusion criteria; IBR not discussed or could not be differentiated from delayed breast reconstruction; patients with ductal carcinoma *in situ*; prophylactic mastectomy; studies that did not clearly state the number of women aged 65 years or more with or without IBR after mastectomy; lack of comparison between older and younger women with and without IBR; review article, editorial or case report; and articles with restricted access.

### Data extraction

Data were extracted by one reviewer using a piloted modified worksheet, including: country, year of study, total number of patients included in study, patient age, and number of patients with and without PMIBR for primary invasive breast cancer. Extracted data were double-checked by a second reviewer.

### Critical appraisal

To assess the studies identified by the search, a system proposed by Harbour and Miller was used^29^. The quality of the studies was evaluated using the PRISMA statement^27^. The level of evidence was assessed as level I–VII using the guide derived by Harbour and Miller^29^. Risk of bias was assessed using the Cochrane risk-of-bias assessment tool^30^, and was done at a study and outcome level.

## Results

### Summary

**Fig. 1:**
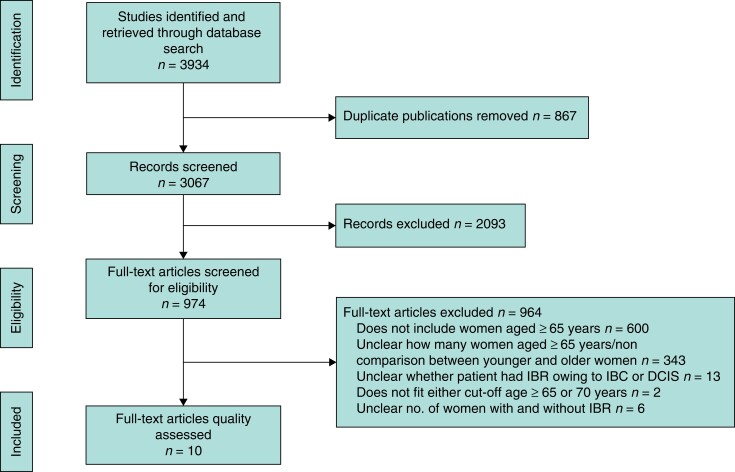
Flow diagram showing selection of articles for review IBR, immediate breast reconstruction; IBC, inflammatory breast cancer; DCIS, ductal carcinoma *in situ.*

A total of 10 studies^31–40^ met the inclusion criteria for this review (*Fig. 1*). These studies were published between 2012 and 2020. In total, 466 134 women who had undergone mastectomy for primary breast cancer were included in these studies.

### General characteristics

Characteristics of the included studies are shown in *Table 1*. Six studies were conducted in the USA, and one study each in the UK, Sweden, Italy, and New Zealand. Most were retrospective studies; there was only one prospective study. A summary of each study is presented in *Table 2*.

**Table 1:** Summary of characteristics of included studies

Reference	Country of study	Study design	LoE	Aim of study	Total no. of women who had mastectomy for invasive breast cancer (*n* = 466 134)	Younger women (aged < 65 years)		Older women (aged ≥ 65 years)	
						IBR for invasive breast cancer (*n* = 142 533)	Without IBR for invasive breast cancer (*n* = 170 765)	IBR for invasive breast cancer (*n* = 15 144)	Without IBR for invasive breast cancer (*n* = 137 692)
**Frisell *et al.*** ^31^	Sweden	R	III	To determine associations between socioeconomic status of patients with BC and IBR rates	3131	250	1353	17	1511
**Heeg *et al.*** ^32^	USA	*P*	II	To investigate whether IBR after mastectomy reduces the likelihood of timely initiation of adjuvant CT	4658	1451	2891	16	300
**Morrow *et al.*^33^**	UK	R	III	To investigate current oncoplastic breast conservation surgical practice in Scotland	1490	361	319	24	786
**Sada *et al.*^34^**	USA	R	III	To analyse postmastectomy complications in women aged ≥ 65 years	1476	873	259	95	249
**Campbell *et al.*^35^**	New Zealand	R	III	To understand factors influencing use of surgical options by New Zealand women with newly diagnosed BC	4204	882	2058	6	1258
**Gibreel *et al.*^36^**	USA	R	III	To investigate impact of age and co-morbidities on use and outcomes of PMIBR	364 767	114 314	135 702	13 187	101 564
**Aurilio *et al.*^37^**	Italy	R	III	To quantify clinical outcomes of patients who received IBR	376	291	51	6	28
**Aurilio *et al.*^38^**	USA	R	III	To investigate whether PMIBR and neoadjuvant CT influenced outcome in patients with ER-negative BC	122	56	62	1	3
**Golshan *et al.*^39^**	US	R	III	To identify processes that contribute to delays from first consultation to first definitive surgery	419	261	110	7	41
**Hershman *et al.*^40^**	US	R	III	To evaluate association between demographic, hospital, physician, and insurance factors and receipt of IBR	85 491	23 794	27 960	1785	31 952

LoE, level of evidence; IBR, immediate breast reconstruction; R, retrospective; BC, breast cancer; *P*, prospective; CT, chemotherapy; PMIBR, postmastectomy immediate breast reconstruction; ER, oestrogen receptor.

**Table 2:** Summary of included papers

Reference	Were reasons for disparity in uptake of IBR in younger and older women explored?	Methods	Factors suggested/reasons hypothesized by authors affecting disparity in uptake of IBR in younger and older women	Conclusion
**Frisell *et al.*^31^**	Yes	Data on tumour characteristics, surgical procedures, and planned oncological treatment were extracted from Swedish National Breast Cancer Register and analysed	Patient-associated factors	Several socioeconomic factors independently influence IBR rates. Patient-reported information and involvement in the surgical decision-making process remain independent predictors for having IBR.
			Younger age	
			Non-invasive tumour	
			No clinically involved lymph nodes	
			Single marital status	
			Physician-associated factors	
			Patient-reported preoperative information	
			Patient-reported involvement in decision-making process	
			System-associated factors	
			Unemployed/retired	
			Higher socioeconomic status	
**Heeg *et al.*^32^**	No	n.a.	n.s.	PMIBR reduced the likelihood of receiving adjuvant CT within 6 weeks, but not within 9 or 12 weeks. Thus, IBR is not contraindicated in patients who need adjuvant CT after mastectomy
**Morrow *et al.*^33^**	No	n.a.	n.s.	Oncoplastic breast conservation occupies its own niche between standard breast conservation and mastectomy
**Sada *et al.*^34^**	No	n.a.	n.s.	Differences in postoperative complication rates after mastectomy with IBR for older *versus* younger patients
**Campbell *et al.*^35^**	No	n.a.	Patient-associated factors	Surgical management of women with localized BC was generally in line with guidelines, but there is potential to further increase the use of breast conservation and IBR in suitable patients
			Increasing age	
			Ethnicity	
			Cancer stage	
			Co-morbidity	
			Having postmastectomy RT	
			System-associated factors	
			Public or private hospital socioeconomic status	
**Gibreel *et al.*^36^**	No	n.a.	Patient-associated factors	PMIBR rates are increasing. Higher 30-day unplanned readmission rates among older women
			Younger age	
			White race	
			Fewer co-morbidities	
			*In situ* cancer	
			Smaller clinical tumour size	
			Clinically negative axillary nodes	
			Well differentiated tumours	
			Postmastectomy radiation	
			Adjuvant and neoadjuvant CT	
			Physician-associated factors	
			Provider recommendation	
			System-associated factors	
			Surgery at an academic/research or comprehensive community cancer programme	
**Aurilio *et al.*^37^**	No	n.a.	n.s.	IBR does not significantly affect prognosis of patients with ER-negative BC
**Aurilio *et al.*^38^**	No	n.a.	n.s.	IBR following total mastectomy in patients with ER-negative disease after neoadjuvant CT is associated with a worse rate of local relapse
**Golshan *et al.*^39^**	No	n.a.	n.s.	Areas to focus on future changes include coordination of same-day plastic surgery visit, realignment of operating room block time to better overlap between two surgeons in case of mastectomy and reconstruction, real-time second-opinion breast imaging, and addition of wire localization slots
**Hershman *et al.*^40^**	Yes	Retrospective hospital-based analysis with Perspective, a voluntary, fee- supported database originally developed to measure resource use and quality of care. Logistic regression analysis was used to determine factors predictive of IBR	Patient-associated factors	PMIBR has increased significantly over time; however, modifiable factors such as insurance status, hospital size, hospital location, and physician volume strongly predict PMIBR
			Increasing age Women of black race	
			Single marital status	
			Increased co-morbidities	
			System-associated factors	
			Insurance status	
			Large hospital size	
			Rural hospital location	
			Non-teaching hospital	
			High hospital volume	
			High surgeon volume	

IBR, immediate breast reconstruction; n.a., not applicable; n.s., not stated; PMIBR, postmastectomy immediate breast reconstruction; CT, chemotherapy; RT, radiotherapy; BC, breast cancer; ER, oestrogen receptor.

### Level of evidence

One study^32^ was rated as providing level II evidence, and nine^31,33–40^ as providing level III evidence.

### Risk-of-bias assessment

The results of risk-of-bias assessment for the included studies are summarized in *Table 3*.

**Table 3:** Summary of risk-of-bias assessment

Reference	Selection bias	Performance bias	Attrition bias	Detection bias	Reporting bias	Other bias
**Frisell *et al.*^31^**	+	−	−	−	−	−
**Heeg *et al.*^32^**	+	−	−	−	−	−
**Morrow *et al.*^33^**	+	−	−	−	−	−
**Sada *et al.*^34^**	+	−	−	−	−	−
**Campbell *et al.*^35^**	+	−	−	−	−	−
**Gibreel *et al.*^36^**	+	−	−	−	−	−
**Aurilio *et al.*^37^**	+	+	−	+	−	−
**Aurilio *et al.*^38^**	+	+	+	+	−	−
**Golshan *et al.*^39^**	+	−	−	−	−	−
**Hershman *et al.*^40^**	+	−	−	−	−	−

+, High risk of bias; −, low or unclear risk of bias. Selection bias: random sequence generation and allocation concealment. Performance bias: blinding of participants and personnel. Attrition bias: incomplete outcome data. Detection bias: blinding of outcome assessment. Reporting bias: selective reporting. Other bias: bias owings to problems not covered elsewhere.

### Disparity in uptake of PMIBR between older and younger women

In total, 466 134 women who had undergone mastectomy for primary breast cancer were included in these studies, of whom 67 per cent (313 298) were younger and 33 per cent (152 836) were older women. Overall, 142 533 of 313 298 younger women underwent PMIBR (45 per cent), whereas only 15 144 of 152 836 older women had PMIBR (10 per cent).

### Factors affecting uptake of PMIBR

Among studies that aimed to identify and explore factors affecting uptake of PMIBR in both younger and older women, only two^31^ specifically aimed to explore underlying reasons affecting the uptake of PMIBR in older women. The factors identified in these two studies can be classified into three main categories: physician-associated factors, patient-associated factors, and system-associated factors.

Eight studies^32–39^ hypothesized reasons for lower uptake of PMIBR in both younger and older women, whereas two^31,40^ analysed data collected to determine factors affecting uptake of PMIBR in older women.

Frisell *et al.*^31^ extracted and analysed data on tumour characteristics, surgical procedures, and planned oncological treatment from the Swedish National Breast Cancer Register. Hershman *et al.*^40^ reported a retrospective hospital- based analysis with a voluntary, fee-supported database originally developed to measure resource use and quality of care. Logistic regression analysis was used to determine factors predictive of IBR. The findings of these two studies can be classified into three overarching categories (*Table 4*); these are explained in more detail below based on the findings from two studies^31,40^ and supported by hypotheses from two others^35,36^. The remaining studies^32–34,37–39^ did not make any attempt to explain their findings.

**Table 4:** Themes of two studies^31,40^ exploring factors affecting uptake of postmastectomy immediate breast reconstruction in older women

Physician-associated factors	Patient-associated factors	System-associated factors
Surgeon’s practices	Patient characteristics	Insurance status
		Income status
		Hospital size/location
	Tumour characteristics	Physician volume
		Hospital size/location
		Physician volume

#### Physician-associated factors

Frisell *et al.*^31^ highlighted the level of surgeon influence on decisions regarding women’s surgery. Younger women were more likely to undergo PMIBR if it was recommended by their surgeons. This was also suggested by Gibreel *et al.*^36^ as a potential explanation for their findings.

#### Patient-associated factors

Two studies^31,40^ reported patient characteristics as a barrier to PMIBR, which was also suggested by two other studies^35,36^. Hershman *et al.*^40^ stated that increasing age of women and patients with more co-morbidities were less likely to undergo PMIBR, which was supported by Campbell *et al.*^35^. It was also hypothesized that having postmastectomy radiotherapy^35,36^, or adjuvant or neoadjuvant chemotherapy^36^ were associated with a reduced likelihood of undergoing PMIBR.

Frisell *et al.*^31^ revealed that single women were more likely to have PMIBR than married women, which was also suggested by Hershman *et al.*^40^. Frisell *et al.*^31^ stated that younger women were more likely to have PMIBR, which was also inferred from two other studies^35,36^. Hershman *et al.*^40^ concluded that white women were more likely to have PMIBR than Maori, Pacific, and black women, which was supported by two other studies^35,36^. Gibreel *et al.*  ^36^ also hypothesized that women with fewer co-morbidities were more likely to have PMIBR.

Frisell *et al.*^31^ reported that more favourable tumour characteristics, such as lower tumour burden and absence of evidence of clinically involved lymph nodes, were associated with increased rates of PMIBR. This was supported by Gibreel *et al.*^36^, who also postulated that smaller tumour size and well differentiated tumours were associated with increased rates of PMIBR.

#### System-associated factors

Two studies^31,40^ reported insurance-related, hospital-related, and income-related factors as reasons affecting uptake of PMIBR, and these findings were also supported by Campbell *et al.*^35^.

Having insurance cover^40^ and higher socioeconomic status^31^ were proven to be predictors of increased rate of PMIBR. Campbell *et al.*  ^35^ also suggested that higher socioeconomic status was related to an increased rate of PMIBR. Being unemployed or retired was associated with a reduced likelihood of undergoing PMIBR^31^. Hershman *et al.*  ^40^ revealed that undergoing surgery in a non-teaching hospital, high surgeon volume, high hospital volume, and large hospital size were associated with an increased rate of PMIBR. Gibreel *et al.*  ^36^ hypothesized that having surgery in an academic/research hospital or comprehensive community cancer programme were associated with an increased rate of PMIBR.

## Discussion

Chronological age alone is an unreliable predictor of cancer treatment benefit and tolerance, and should not influence treatment decisions surrounding breast reconstruction^1^. Both older and younger women want to be offered different reconstructive options. Bowman *et al.*^8^ carried out a survey of 75 women, with a response rate of 81 per cent (61 patients), of whom 31 had delayed reconstruction. However, only 16 per cent of older patients who had delayed breast reconstructions stated that the option of immediate reconstruction was presented to them at the time of diagnosis. Among those who were not told about immediate reconstruction, 100 per cent felt that it should have been discussed with them^8^. In a study from the UK^41^, older women reported that they would have liked to discuss breast reconstruction options, but felt that the lack of discussion with their surgeons was attributed to their age. All members of the healthcare team need to be educated on the importance of shared decision-making, and information available should be widely accessible to meet the needs of the older patient, for example if visual or hearing impairments are present.

Compared with younger women, older women have different tumour characteristics, physiology, social dynamics, and priorities at this stage in their life^42–44^. Hence, older women face unique challenges specific to each phase of breast cancer treatment and receive different breast cancer treatments^44^. It is crucial that each woman receives individualized treatment for breast cancer. Older women generally have more co-morbidities than younger women, but the severity of the disease may differ between older women. A review by James *et al.*^26^ concluded that a minority of older women are likely to accept reconstructive surgery, but those who do have outcomes that are at least as good as those in younger patients, and experience good quality of life. This paper was not included in the present review. Even though James *et al.*^26^ included patients aged 65 years or older, the study did not clearly state the number of women in this age group with or without IBR after mastectomy, and so did not meet the inclusion criteria for the present review. Furthermore, this systematic review was excluded because the individual studies should be picked up by the search strategy.

In recent years, a few studies ^45–47^ have summarized trends showing gains in life expectancy in women. Women with breast cancer have longer survival rates compared to the past^48–50^. It is important to identify the needs of older women through the use of a geriatric screening tool to determine their functional age and optimize their care^51^. The G8 screening tool was developed for older patients with cancer; this encompasses patients’ food intake, weight loss, mobility, neuropsychological problems, BMI, medications, and self-perception of health^52^. It is not time-consuming to complete and identifies older women who would benefit from a detailed assessment^52^.

The UK National Audit of Breast Cancer in Older Patients (NABCOP) 2021 annual report^25^ clearly demonstrated that women aged 70 years and over were less likely to receive PMIBR and standardized breast cancer treatment than younger women. Although the reasons behind this variation were multifactorial, the presence of co-morbidities was an important influencing factor. The report recommended using the NABCOP fitness–frailty assessment form, which consists of sections including the Abbreviated Mental Test Score, three screening questions on medical or cognitive co-morbidities, and the Clinical Frailty Scale. This aimed to provide a standardized measure of patient’s fitness for surgery as part of a holistic assessment. This report was not included in the present review. Even though the report included patients aged 50 years and above, the study did not clearly state the number of women aged at least 65 years with or without IBR, and therefore did not meet the present inclusion criteria.

One method of detailed assessment of geriatric conditions is comprehensive geriatric assessment (CGA). CGA is a multidisciplinary process focused on evaluating an older patient’s parameters of physical function, co-morbidity, social support systems, cognitive function, psychological status, nutrition, and medication^53,54^. It is highly recognized in oncology, with the International Society of Geriatric Oncology and the National Comprehensive Cancer Network recommending its incorporation into cancer treatment planning^55^. Current literature on CGA is mainly related to cancer surgery and oncological therapies such as administration of chemotherapy. It has been suggested that CGA is an important factor in determining treatment and early management of early breast cancer^56^, but there is currently a lack of evidence showing its usefulness in guiding the decision-making process for breast reconstruction, which is not a direct cancer treatment. However, breast reconstruction is an integral part of modern breast cancer care aimed at improving the patient’s quality of life and function, which is also aligned with the aims of cancer surgery. Introducing CGA into breast clinics may assist surgeons in assessing older women’s fitness for treatment and the appropriateness of treatment^56^. This will help surgeons develop a personalized approach to assessing older women’s suitability for different types of breast cancer treatment and breast reconstruction options, and maximizing their quality of life^56^.

This review has highlighted that surgeons play a critical role in determining whether a woman undergoes PMIBR. Patient-reported information and involvement in the decision-making process regarding breast reconstruction were strong predictive factors for IBR^57–59^. ‘Surgeon strong recommendation’ was reported by about 92 per cent of women as a reason for undergoing PMBR^60^. Surgeons may play an even larger role in the treatment decision-making of older women, who were described as relying heavily on the advice of healthcare professionals as they felt that treatment should be left to surgeons who had specialist knowledge^61^. By encouraging surgeons to provide more information and discussing reconstructive options to older women at diagnosis, they may be more empowered to choose reconstruction.

Interestingly, many studies have shown how surgeons’ implicit bias regarding age and ethnicity contributes to health disparities^6,62,63^. Both increasing age and black race have previously been associated with lower rates of PMIBR. Black women were overall less likely to undergo PMIBR than white women across all age groups. This racial disparity remains even after accounting for patients’ insurance status^64^. It is crucial that surgeons recognize their susceptibility to implicit bias as this affects surgical practice patterns, which may be prejudicial to patients.

In the UK, although there is free universal access to the National Health Service (NHS), there is substantial regional variation in uptake of PMIBR in England depending on a patient’s residential address, known as postcode lottery. Some hospitals may not offer plastic surgery or oncoplastic breast surgery services. As such, patients may need to travel a long distance to a different NHS Trust to gain access to reconstructive surgeons at hospitals with those services. This may be off-putting to older patients who may rely on relatives or public transport to attend hospital appointments. This potential barrier should be recognized and discussed frankly with the patient when discussing treatment options.

Older women are often excluded or under-represented from major studies of breast reconstruction^24^. A multitiered approach is needed to raise awareness that PMIBR is a safe procedure in older women, with studies^18,65^ showing that PMIBR can improve the quality of life of older women as much as younger women, and to challenge the preconception among healthcare professionals that older women do not want breast reconstruction^24,66,67^. Older women should be supported to participate in clinical trials and research studies.

Multiple studies have reported that the uptake of PMIBR is significantly lower in some ethnic groups^68,69^. African American, Hispanic, and Asian women were 52, 55, and 71 per cent respectively less likely to undergo PMBR than white women^70,71^. A potential explanation for this is that community and cultural values largely influence the uptake of PMIBR. For instance, PMIBR is considered as an elective cosmetic procedure in the Asian community^2^. The presence of co-morbidities^11^ and change in perception of body image with ageing may also explain why some older women are less likely to receive IBR than younger women^2^. Ideally, all reconstructive options (both immediate and delayed) should be discussed with all patients, both older and younger women, so that they can make an informed decision about which is best suited to their breast cancer treatment, as well as personal and cultural beliefs.

This review has quantified the disparity in uptake of PMIBR between younger and older women. Its biggest limitation is that it included a small number of articles, and only two studies exploring factors affecting the uptake of PMIBR in older women. In addition, this review is based on retrospective data, and as such may be subject to selection bias. The population of older women who received PMIBR and were included in the studies might have had favourable factors, such as higher socioeconomic status, higher level of education, fewer co-morbidities, and more favourable tumour characteristics than older women who were not included.

Finally, most of the studies were conducted in the USA. The data may not be extrapolated accurately to other countries, where the experiences of older women may be very different. Country-specific research would be helpful, as nuances related to the medical system, but also larger societal issues surrounding race and class, may have ramifications for the patient experience. Individualized breast cancer treatment discussions to improve the uptake of PMIBR in older women are supported by recommendations^72^ of the European Society of Breast Cancer Specialists and the International Society of Geriatric Oncology.

## Supplementary Material

znac251_Supplementary_Data
