# Supplementary figures and images for: Immediate breast reconstruction uptake in older women with primary breast cancer: systematic review
Source: Br J Surg. 2022 Aug 1;109(11):1063–72. doi: 10.1093/bjs/znac251 (PMC10364779; doi:10.1093/bjs/znac251)

**Appendix S1:** Search strategy for Medline


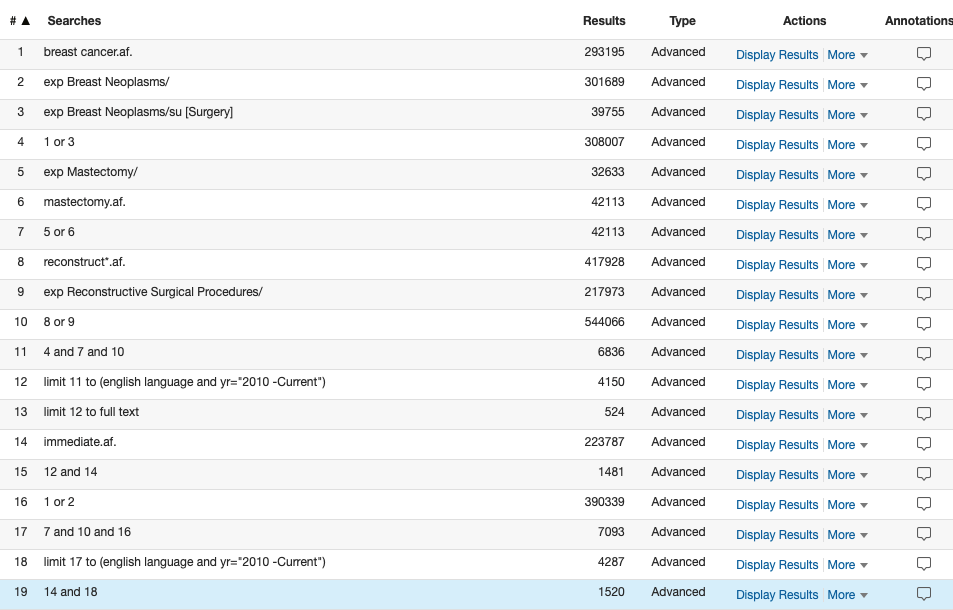


Search strategy for Embase


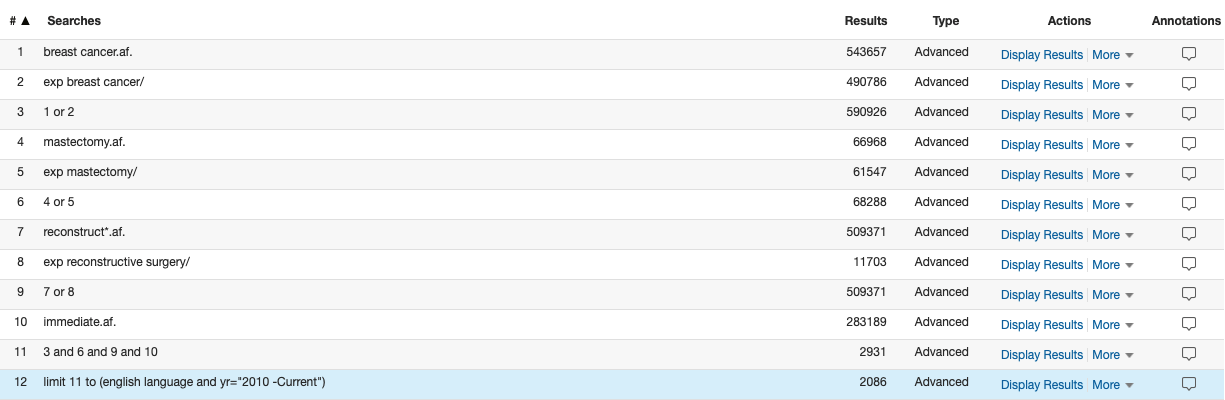


Search strategy for Pubmed


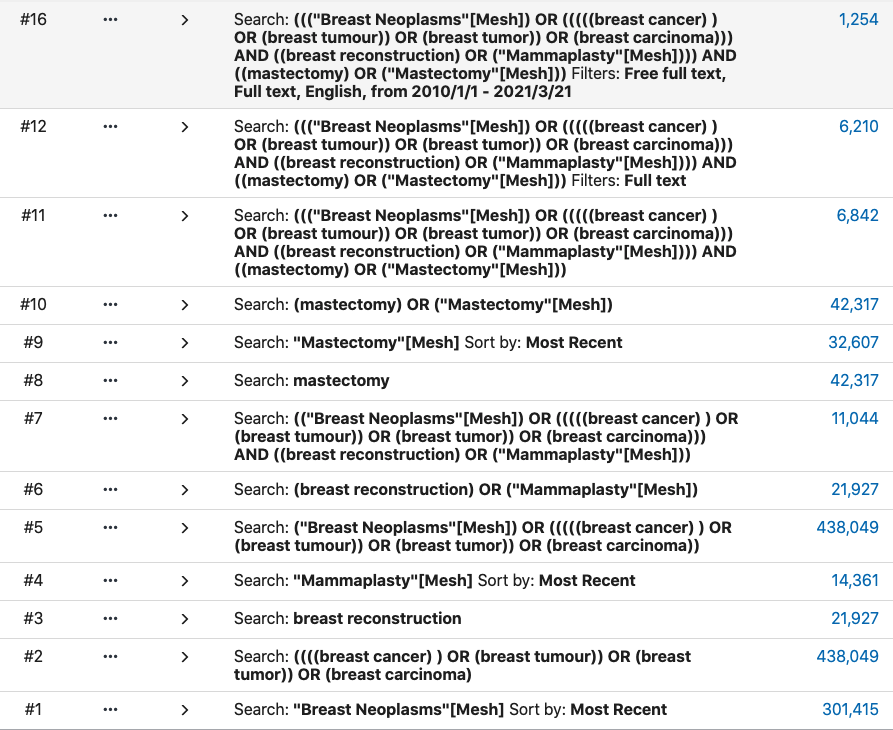

Supplement: znac251_Supplementary_Data [file znac251_supplementary_data.zip › Supplementary_Appendix_1.docx]
